# Ethyl Bromide as an Anæsthetic

**Published:** 1899-04

**Authors:** E. B. Dickinson

**Affiliations:** Amherst, Mass.


ARTICLE IX.

### ETHYL BROMIDE AS AN ANÆSTHETIC.

E. B. DICKINSON, D. D. S., AMHERST, MASS.

I desire that you will not confound ethyl bromide with ethyl chloride, ethyl, or any of the bromides used as local anæsthetics.

Nunnely, of Leeds, was probably the first to utilize the analgesic properties of ethyl bromide, and to advocate its use as an anæsthetic.

Turnbull, of Philadelphia, was much interested in it, and from 1878 to 1882 spent considerable time in investigating its action. At about this period many physicians and dentists used this means of abolishing pain, and from Philadelphia its use spread in various directions. Dr. Price speaks of it in the St. Louis "Medical and Surgical Journal," vol. XLV, p. 297. The great difficulty then seems to have been to obtain a perfectly pure article, and this is scarcely yet overcome, on account of the impurities of the drug and methods of administration, regardless of its physiological properties.

The advantages to be derived by its use were by no means so great as they should have been, and perhaps this fact, conjointly with that of the difficulty of obtaining a supply promptly, would explain its limited use until recently, in spite of its indorsement by such authorities on anæsthesia as Turnbull in the United States, Richardson in England, and Dartee in France. Ethyl bromide is a colorless liquid, considerably heavier than water, boiling at 39 degrees C., a temperature slightly over that of the body. The liquid is non-inflammable, has an ethereal odor a sweetish taste, and possesses marked properties of local anæsthesia produced by contact, as well as the general properties of analgesia with consequent anæsthesia when inhaled. Ethyl bromide should have a perfectly neutral reaction with reference to litmus paper. It should be kept protected from the air and from actinic rays, for although not so easily altered as chloroform, it is nevertheless subject to decomposition unless such precautions are taken.

No ethyl bromide should be used if there can be any doubt of its purity. Let me say right here, I have had samples tested bearing the marks of some of the prominent pharmaceutical chemists of this country, and have found impurities which condemn its use as an anæsthetic.

I can indorse a preparation put up by Dr. Goesmann, having used it myself and knowing that all his products are tested physiologically before leaving the laboratory. The impurities of ethyl bromide may come either from the process of manufacture or from decomposition.

Among those found are free bromine, acetone, and phosphorus, in combined form. Some of the samples that I have seen have been even yellow and some showed a deposited sediment in the bottom of the bottle. Absolute purity must be assured, then safety and the best results are practically certain. As a caution, note carefully that no other organic bromide be furnished in place of ethyl bromide. Too many fatal errors have been committed by a substitution of similarly named substances, through the criminal ignorance or carelessness of the pharmacist.

Its physiological properties, quoting from Dartee, von Riemacki, Malherbe. Applied to the skin or mucous membrane, ethyl bromide has a slight vesicating effect; the skin reddens to a perceptible degree, and is warmer to the touch than the surrounding parts not subjected to treatment. In a few minutes the phenomenon of local anæsthesia appears, probably induced, as far as the skin is concerned, by the rapid evaporation of the ethyl bromide and consequent congelation of tissue, in manner similar to that of ether when applied locally with a Richardson pulverizer. The internal administration of ethyl bromide is accomplished to best advantage by its inhalation, and in this manner it has been administered in all experiments for the study of its physiological action as hereafter described.

Ethyl bromide apparently effects a chemical union with the iron salt of the red corpuscle of the blood, to which we attribute the function of transporting oxygen. The ethyl bromide, when inhaled, seems to so unite with the blood as to prevent its taking up the oxygen, and to that extent becomes an agent of suffocation similar in action to that of carbon monoxide. If this fact be borne in mind, it will be readily understood why a short administration of the anæsthetic, even in considerable quantity, but interrupted by periods allowing of full respiration, would portend no danger to life, whereas a continued and protracted inhalation of this same substance might so saturate the blood as to reduce its oxygenating function to a degree dangerous and perhaps to a point beyond recovery. This fact seems to have been ignored by the early users of this anæsthetic, who gave it by a continuous method, such as is used for chloroform and ether. The action of ethyl bromide upon the nervous system is one of great rapidity, but in clearly defined stages. A very slight inhalation dilates the capillaries, probably by action on the vasomotor, producing profuse perspiration, and stimulates all the secretory glands.

The pheripheric nervous paralysis evinced by the congestion of the capillaries is now followed by the regular symptoms of an ascending nervous insensibility. The sense of touch still remains, but of an analgesic nature that is to say, that, although there is perfect knowledge of the touch, there seems to be no appreciation of the force applied, the irritability of the skin totally disappears, and such a thing as a pin prick is only felt as a contact, not at all as a pain. The spinal reflex action determined by peripheric excitement is now abolished, and this phenomenon rises higher up the spinal ganglion.

It is particularly instructive, in experimenting with animals in which these nervous changes are accomplished with sufficient slowness, to observe how the motor reflex remains normal long after the peripheric sensibility is lost; that is to say, when the animal has reached this stage, it remains immovable when pricked or pinched, but if frightened by a rapid gesture, or by a noise, immediately struggles, its members endowed with perfect co-ordinate motion. The same phenomenon is easily seen in the human subject when in this stage of analgesia, for while it is then possible to perform an absolutely painless operation, it is still perfectly possible to maintain a conversation with the patient, or that which is still more easy and fully as striking, to command the subject to execute any motion with a member and it is immediately done, proving the maintenance of full control over the motor reflexes in spite of the loss of all peripheric sensibility and dependent reflexes.

As the peripheric nerve paralysis affects the corresponding reflex centre higher and higher up the cord, at a given time it is manifest that the cervical origins of the pneumogastric nerve are affected, and the respiration is for a moment arrested by a voluntary action of the subject, thus evincing the consciousness of the invasion of an exterior influence into this purely reflex centre. The shock of invasion provokes the voluntary arrest of respiration, which is only momentary. The breathing is at once renewed, but is now deep and totally diaphragmatic, slow but regular. The affection of the pneumogastric centre is immediately accompanied by the phenomena of ocular reflex, evinced by the paralysis of the iris and its relaxation and consequent dilatation of the pupilla. At this moment a slight clonic muscular reaction is possible and the lines of the face harden, evincing the setting of the muscles in a general contraction throughout the body. Such a phenomenon is of a very slight degree and of slight persistence. There is no convulsive action, and up to this point no trace of any effect on the central nervous system is manifested. And this is what is to be expected in a case of an ascending reflex paralysis such as is developed by ethyl bromide. The action of the drug is now reaching the bulbus, and at this point its effect on the central nervous system commences. The pupil is dilated to its maximum, and there is no reaction to light; the eyes eyes are fixed, their optical axes parallel, as a general rule; the respiration is deep, the cardiac beat slightly quickened and strong; peripheric congestion. There has been some ringing in the ears. At such a point the patient loses the power of the motor nerves, ceases to hear commands, becomes unable to obey them by a process of invading lassitude, and with a possible instant of mental excitement drops into unconsciousness, the last phenomenon of a complete anæsthesia induced by ethyl bromide. If at this moment the administration of the drug is stopped, the unconscious condition persists only for a few minutes—three to seven—then gives way to a lethargic condition in which a patient hears and understands what it said, and may feel the contact of an instrument. Little by little he reaches a condition in which strong commands will obtain a motor obedience, and soon will come a reply to questions, but for some moments there is no perception of pain, and this sense is but slowly re-established and fully regained only at the lost phenomenon of recovery from the drug. As consciousness returns the phenomena noted pass in inverse order, for we are dealing with the retrograde nervous tension, the return to a normal condition emanating from the central nervous system, down the spinal reflex centre, through the sensory reflexes to the periphery, and at that point reaching the complete recovery to the normal condition. It is to be remembered that the motor reflexes are not lost until unconsciousness appears, but their recovery is slower than that of the central nervous system, for the reason that the drugged sensory nerves shut out all impressions, and until their recovery the central nervous system will not call into action its motor nerves. It is of exceeding interest to observe this phenomenon. Suppose that a patient is recovering from unconsciousness while the extraction of teeth is going on, there will be no motion made by the subject, unless such motion be commanded by the operator, until a return to the normal condition is so complete that the sense of pain (peripheric sensation) provokes a motor reflex. It is remarkable how long an interval there is between the possibility of exciting the motor reflex by an auditory sensation and that of obtaining any motion in response to the sensation of pain.

A review of experiments, the substance of which is outlined above, decided von Ziemacki to place in parallel columns, for purpose of comparison, some of the phenomena induced by ethyl bromide and those evinced when chloroform is used as an anæsthetic. Such a tabulated form of statements is very striking, and is worth repeating. The conclusions are in substance as follows :

1. Chloroform acts on the intelligence, which becomes troubled; sensation of touch and pain remains unaltered. Ethyl bromide first acts on the sensation of pain; analgesia complete first of all; leaves the intelligence almost intact.

2. Chloroform causes strong excitation of the central nervous system. Ethyl bromide causes no wandering of the mind, no excitation of the central nervous system.

3. Following the chloroform sleep is the relaxation of the muscles. Following ethyl bromide, a slight tonic reaction of the muscles.

4. Under chloroform then commence, successively, the weaking alteration and loss of consciousness. Under ethyl bromide, after the analgesia the consciousness is lost.

5. Chloroform acts last of all on the sense of pain. Ethyl bromide acts last on the intelligence.

6. Chloroform does not stimulate the respiration during narcosis. Ethyl bromide stimulates respiration during narcosis.

7. Chloroform never causes any clonic contraction. Ethyl bromide sometimes produces clonic contraction during narcosis.

8. Chloroform causes nausea and vomiting almost invariably after narcosis. Ethyl bromide is rarely followed by sickness or vomiting.

9. Recovery from chloroform depression and return to consciousness are so slow to appear as is the anæsthesia slow to induce. The anæsthesia and its recovery are both rapid when induced by ethyl bromide.

Malherbe, in terminating his report on the physiological action of ethyl bromide, concludes as follows : "The action of the bromide is much less toxic than that of chloroform; the dilatation of the pupil and contraction of the masseters appear rapidly. The respiration, slow for an instant, increases in rapidity and finally becomes irregular if the dose is prolonged and heavy. The muscular contraction disappears with the suspension of the anæsthetic inhalation. The reagent determines a strong excitation of the excretory glands. Ethyl bromide seems to act as a stimulant on the nervous system, and particularly the vital centres. The respiration is threatened more than the heart.

"PREPARATION AND ADMINISTRATION.—Place the patient in as nearly a reclining position as possible. See that the neck and waist-bands are free, so that the patient can take a full, deep inhalation easily; the stomach empty, that is not essential. Place a few drops of the ethyl bromide on a folded towel (one of large mesh preferred) and pass it over the nose of the patient to accustom to the odor. Immediately, upon the upper side of the towel, pour out about three grammes of the reagent, and rapidly reverse the towel; apply it closely to the nose and mouth so that every inhalation may be taken through its meshes.

"For a moment the patient holds his breath, perhaps makes a slight effort to pull away the towel, at the same time swallowing in rapid succession the saliva which is secreted abundantly. In an instant a long inhalation is made, followed by others, especially so if commended by the operator. The face becomes red, eyes fixed, eyelids difficult to raise with the finger, lines of face drawn, jaw set, and a general muscular contraction of short duration may be manifested. After five or six inhalations the patient has lost all sense of pain, but is still conscious. At this point we could begin operating could we continue the anæsthetic. This is the time to begin operations outside of the mouth. A few grammes again poured on the towel and inhaled result in complete unconsciousness, if not already obtained with the first dose. Time, one-half to one minute and a half, seldom longer. Pulse rapid and strong, respiration deep and of about normal rapidity. The longer the drug is applied the longer till recovery.

"For minor surgery, extracting teeth or nerves, opening abscesses, etc., I know of no better anæsthetic. Easily applied, quick in action and in recovery. No scenting up of office, or hours spent in the administering and recovery of the patient from the nausea and vomiting generally accompanying ether or chloroform.

"As to the safety of the reagent, in investigating statistics where pure ethyl bromide alone has been used I find no fatalities, bad results, or after-effects. In my own experience, having administered it to both old and young, people feeble in health as well as the robust, I have met with only pleasing results, and I find that patients take very kindly to it. Let me commend ethyl bromide not only to your notice, but your application."—*Int. Dental Journal.*

